# Sex estimation in a Turkish population using Purkait’s triangle: a virtual approach by 3-dimensional computed tomography (3D-CT)

**DOI:** 10.1080/20961790.2021.1905203

**Published:** 2021-04-27

**Authors:** Julieta G. García-Donas, Suna Ors, Ercan Inci, Elena F. Kranioti, Oguzhan Ekizoglu, Negahnaz Moghaddam, Silke Grabherr

**Affiliations:** a Centre for Anatomy and Human Identification, School of Science and Engineering, University of Dundee, Scotland, UK; b Department of Radiology, Bakirkoy Training and Research Hospital, Istanbul, Turkey; c Department of Forensic Science, University of Crete, Crete, Greece; d University Center of Legal Medicine Lausanne-Geneva, Lausanne, Switzerland; e Unit of Forensic Imaging and Anthropology, University Center of Legal Medicine Lausanne-Geneva, Lausanne, Switzerland; f Swiss Human Institute of Forensic Taphonomy, University Center of Legal Medicine Lausanne-Geneva, Lausanne, Switzerland

**Keywords:** Forensic sciences, forensic anthropology, sex estimation, computed tomography, Purkait’s triangle

## Abstract

Sex estimation is considered one of the first steps in the forensic identification process. Morphological and morphometrical differences between males and females have been used as means for morphoscopic and metric methods on both cranial and postcranial skeletal elements. When dry skeletal elements are not available, virtual data can be used as a substitute. The present research explores 3-dimensional (3D) scans from a Turkish population to test a sex estimation method developed by Purkait (2005).

Overall, 296 individuals were used in this study (158 males and 138 females). Purkait’s triangle parameters were measured on computed tomography (CT) scans obtained from both right and left femora of each patient at the Bakirkoy Dr. Sadi Konuk Training Research Hospital (Istanbul, Turkey). Intra- and inter-observer errors were assessed for all variables through technical error of measurements analysis. Bilateral asymmetry and sex differences were evaluated using parametric and non-parametric statistical approaches. Univariate and multivariate discriminant function analyses were then conducted.

Observer errors demonstrated an overall agreement within and between experts, as indicated by technical error of measurement (TEM) results. No bilateral asymmetries were reported, and all parameters demonstrated a statistically significant difference between males and females. Fourteen discriminant models were generated by applying single and combined parameters, producing a total correct sex classification ranging from 78.4% to 92.6%. In addition, over 67% of the total sample was accurately classified, with 95% or greater posterior probabilities.

Our study demonstrates the feasibility of 3D sex estimation using Purkait’s triangle on a Turkish population, with accuracy rates comparable to those reported in other populations. This is the first attempt to apply this method on virtual data and although further validation and standardisation are recommended for its application on dry bone, this research constitutes a significant contribution to the development of population-specific standards when only virtual data are available.

**Key points**
- CT analysis using Purkait’s triangle is a suitable tool for assessment of sex in unidentified individuals.
- The best overall estimation rate was achieved with the F11 model, with around 92% of accuracy.
- The results suggested 78.4% to 92.6% correct sex identification rates.
- More research is needed to expand the sample set and verify the results.

## Introduction

The forensic identification process requires relevant information to be gathered from skeletal remains to construct the biological profile of an unknown individual in a medicolegal context [1]. The recording of biological markers supports the categorisation of the unidentified subject into specific groups that account for basic information such as sex, age-at-death, ancestry, and stature. Positive identification of a specific individual entails a process consisting of comparing specific anatomical features with antemortem records with a thorough evaluation of the observations and evidence obtained by the expert [2].

The forensic anthropologist works with skeletonised or partially skeletonised remains to generate a biological profile aiming to assist with positive identification of the unknown individual. Commonly, one of the first steps in the identification routine is sex estimation. Correct sex estimation excludes about half of the general population, making it an imperative step for applying other means of identification that require knowledge of the individual’s sex [3, 4].

The foundations of forensic sex estimation methods are based on a phenomenon known as sexual dimorphism, the morphological and metric differences between males and females of the same species. Those specific sexual variations can be used as a means of differentiation between the sexes [5]. Metric differences using cranial, dental, and postcranial skeletal elements have been used to investigate sexual dimorphism in modern humans through the development of forensic anthropology sex estimation methods [6, 7]. The accuracy of classification depends on the statistical analysis performed, the population of interest, and the bone used for analysis [8]. The cranial and pelvic bones are the most observed skeletal elements. Yet, the femur has also gathered much attention because of its robust nature and high rate of preservation from taphonomic alterations. Moreover, if fragmented, different segments of the femur can be easily identified if anatomical landmarks are present, reconstructed, and used for sex estimation. This results in various levels of accuracy as shown by previous studies examining different femoral segments and different populations [9, 10].

In 2005, Purkait developed a sex estimation method based on a population from Central India using the proximal segment of dry femoral bones [11]. This highly robust bone area is the weight bearing point for the upper body and is expected to be larger in males compared with the relatively lighter axial skeletal weight of females. Other authors have tested and investigated Purkait’s method on Spanish, Greek, European, and African American samples to develop population-specific standards and validate the reliability of the technique [12–14]. Some studies have even applied a variation of the method to femoral radiographs, reporting very satisfactory results [15, 16].

Interpopulation variation for Purkait’s triangle has been reported [12], setting a precedent for further exploration of applying it for sex estimation. Here, we applied this method to a modern Turkish sample, making this the fifth study to use Purkait’s triangle and the first one to utilize virtual data. This work provides a new population-specific method to estimate sex in Turkish individuals by applying Purkait’s triangle, allowing the practitioner to consider its use if the specific case is suspected to have close proximity to the population under study.

## Materials and methods

### Subjects

The sample consisted of computed tomography (CT) scan images collected from patients who consulted the Bakirkoy Dr. Sadi Konuk Training Research Hospital’s clinics (Istanbul, Turkey) between 30 April and 30 July 2015 with official institutional approval and patients’ written informed consents. Multidetector computed tomography (MDCT) scans of 322 individuals who were admitted to the hospital with pelvic diseases were assessed. All participants were required to be at least 18 years of age and have no indication of any pathological condition, deformation, previous surgery, or condition that could affect the normal anatomy of the proximal femur. Thus, 22 patients with pelvic trauma, one patient with congenital deformities, and three patients with acute pelvic deformities, such as avascular necrosis of the femoral head, were excluded. Lower abdominal and pelvic CT images were assessed retrospectively, and all medical documents and patient age and sex were obtained from the hospital data processing centre. Ethics and protocol approval for this study was granted by the Hospital Clinical Research Ethics Committee of Bakirkoy Sadi Konuk Training and Research (19 February 2016).

### Image acquisition

MDCT examination was obtained using a 40-row MDCT scanner (Siemens Medical Solution, Erlanger, Germany). A routine pelvic and abdomen CT protocol was followed with 1 mm slice thickness in the supine position. Tube voltage was 120 kV and effective mAs were varied according to CARE Dose4D (Siemens Medical Solution). All images were transferred to a commercially available workstation and row data were reconstructed using bone algorithms. Three-dimensional (3D) reconstructions of the femo­ral proximal diaphysis for data collection were obtained with the Synapse PACS System (Fujifilm Medical Systems, Lexington, MA, USA). Observers used a manual segmentation method. Age, sex, and all other patient information were concealed from the observers using the “hide data” feature of the software. They were also blind to the patients’ sex and age during data collection.

### Collection of metric data

**Figure 1.**
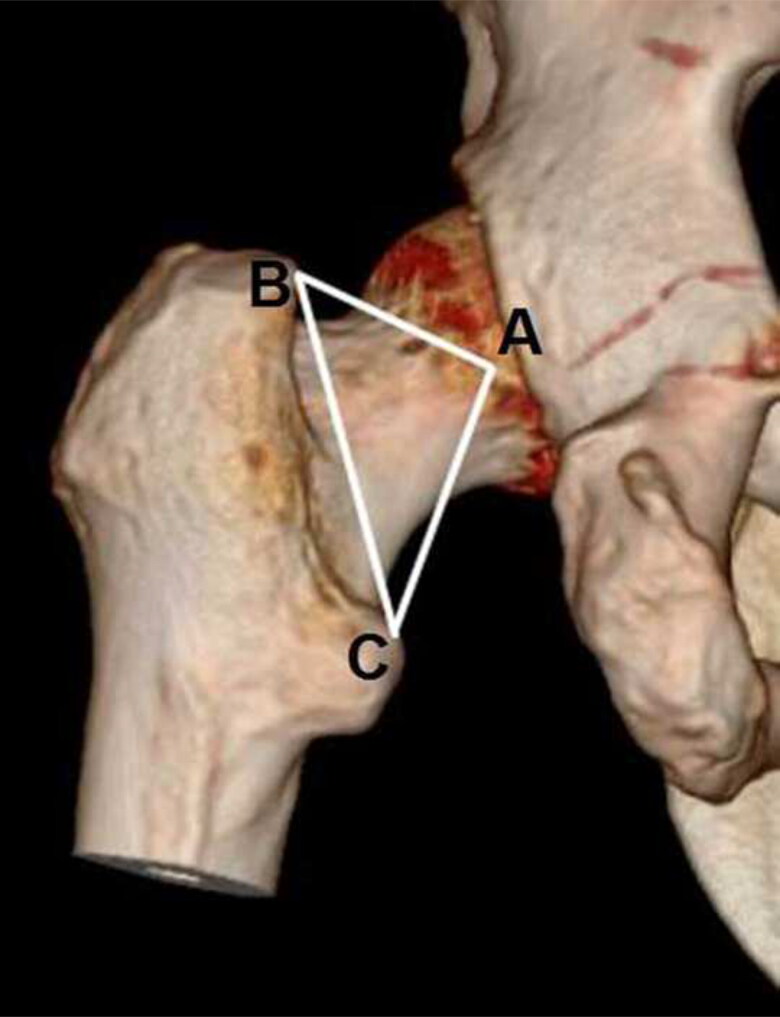
Purkait’s triangle landmarks taken from a CT-scans sample. Point A: The most lateral projecting landmark of the articular facet on the femoral head, Point B: Corresponds to the most medially projecting point of the greater trochanter, and Point C: The most posteromedial point of lesser trochanter.

The parameters defined by Purkait were collected to the nearest millimetre from the right and left virtual femora following the descriptions included in the original method [11]. Purkait’s triangle is defined by three landmarks on the proximal femur. Point *A* is the most lateral projecting landmark of the articular facet on the femoral head, point *B* corresponds to the most medially projecting point of the greater trochanter, and point *C* is the most posteromedial point of lesser trochanter. For detecting point *A*, we used the 3D software of the Synapse PACS System, which allows the user to cut the 3D reconstructed images to discard the acetabular roof and accurately observe the lateral projecting point of the femoral head. Following this protocol, the lengths of lines *AB*, *BC*, and *AC* were measured (Figure 1).

### Statistical analysis

Observer errors were calculated through technical error of measurement (TEM) analysis to explore the reproducibility and reliability of the method on 20 cases randomly selected from the database [17]. Intra-observer error was performed within a 2-month interval. Both observers were highly experienced with the use and manipulation of CT scans for musculoskeletal radiology and forensic anthropology methods. Observers were supported by an experienced forensic pathologist at all stages.

The data were examined for normality using the Shapiro-Wilk test, skewness, and kurtosis values, and graphical representations such as histograms, box plots, and Q-Q plots. Descriptive statistics were calculated for males, females, and total sample. Right and left femoral measurements were explored to assess whether bilateral asymmetry was present. Statistically significant differences between right and left sides were examined through parametric or non-parametric approaches, depending on compliance with test assumptions. Differences between sexes were assessed to examine whether sexual dimorphism exists between the proximal femoral parameters using the Mann-Whitney *U* test. The Sexual Dimorphism Index (SDI) for each parameter was calculated according to Ricklan and Tobias [18] following the following formula: ((*Male mean – Female mean*)/*Male mean*) × 100.

Discriminant function (DF) analysis is a multivariate statistical procedure that allows for making distinctions between groups and generating models that can predict group membership based on a single continuous independent variable or set of continuous independent variables. The classification procedure consists of each case being assigned a discriminant score that is compared with the sectioning point generated for that specific model [19–21]. Both original and cross-validated classification accuracies, the number of individuals, and percentage of correct group membership were provided to evaluate the predictive power and fit of the generated models [20]. Posterior probabilities for the correctly classified cases were calculated and the overall percentage of correct classification for males, females, and total samples for each generated model were reported [22].

Firstly, the assumptions for DF analysis were veri­fied to determine if the data were suitable for using this statistical approach [23]. Once the assumptions were met, we generated univariate DF sex prediction equations. Secondly, multivariate DF analysis was performed to examine all possible combinations of variables. Different sets of variables were manually entered to meet the possible scenarios in which either the right or left femur was present, and all landmarks were intact. Thirdly, DF formulae were developed for the hypothetical scenario for which different states of preservation were considered, such as some of the landmarks (A, B and/or C) not being available. Lastly, a stepwise DF approach using the default F values (F to enter = 3.84, F to remove = 2.71) was applied to automatically select the best combination from all parameters. For all generated models, the “leave one out” classification procedure was applied to understand the stability of the DF models through cross-validation [23]. Data were subjected to statistical analysis using IBM SPSS 24 (Armonk, NY, USA).

## Results

The final sample set contained 296 adult individuals with a mean age of 46.79 years (SD = 18.27). This included a total of 158 males and 138 females with mean ages of 46.53 years (SD = 17.38) and 47.09 years (SD = 19.29), respectively, from which both right and left femoral measurements were collected. A total of 592 measurements were performed.

Observer error results assessed through TEM analysis are presented in Table 1. The variance of error reported within the same observer indicates high reliability of the parameters, with rTEM under the 5% level of acceptance and R indicating 1%–8% of error attributed to measurement error. Inter-observer errors were slightly higher than intra-observer errors, although all parameters demonstrated high reproducibility with rTEM being under 5%. The only parameter drastically over the 95% threshold for the R value was left *BC*.

**Table 1.** TEM, rTEM, and *R* intra- and inter-observer errors for the variables included in this study.

Parameters	Intra-observer error			Inter-observer error		
	TEM	rTEM	*R*	TEM	rTEM	*R*
*Right AB*	0.35	1.10	0.98	0.50	1.57	0.97
*Right AC*	0.45	0.98	0.99	1.28	2.78	0.93
*Right BC*	0.95	1.56	0.92	0.98	1.60	0.92
*Left AB*	0.55	1.72	0.97	0.75	2.33	0.95
*Left AC*	0.51	1.09	0.99	0.97	2.08	0.96
*Left BC*	1.06	1.74	0.92	1.43	2.36	0.86

TEM: technical error of measurement; rTEM: relative TEM.

Descriptive statistics for the samples in this study are presented in Table 2. The results for each sex group indicate that males presented higher values compared with females for both right and left parameters.

**Table 2.** Descriptive statistics for males, females, and total samples.

	Side	Min(mm)	Max(mm)	Mean(mm)	SD	95%CI
Males (*n* = 158)						
AB	R	26.1	39.9	32.10	2.61	31.69–32.51
	L	25.1	39.1	32.16	2.64	31.74–32.57
BC	R	53.4	69.2	62.20	3.41	61.66–62.73
	L	53.9	70.1	62.36	3.45	61.86–62.90
AC	R	34.6	58.1	49.98	3.87	49.37–50.58
	L	34.8	57.8	50.06	3.73	49.47–50.64
Females (*n* = 138)						
AB	R	23.4	35.2	28.26	2.21	27.88–28.63
	L	22.3	34.2	28.35	2.23	27.97–28.72
BC	R	45.7	63.9	53.43	3.19	52.89–53.97
	L	45.8	63.8	53.46	3.13	52.93–53.98
AC	R	33.5	55.1	43.23	3.21	42.69–43.77
	L	33.8	54.2	43.36	3.12	42.83–43.88
Total (*N* = 296)						
AB	R	23.4	39.9	30.31	3.10	29.95–30.66
	L	22.3	39.1	30.38	3.11	30.02–30.73
BC	R	45.7	69.2	58.12	5.49	57.48–58.74
	L	45.8	70.1	58.21	5.54	57.57–58.84
AC	R	33.5	58.1	46.83	4.91	46.27–47.39
	L	33.8	57.8	46.93	4.81	46.38–47.48

*N*: total sample; *n*: sex subsample; SD: standard deviation; CI: confidence interval; r: Right; l: Left.

Preliminary analysis of the data indicated that some of the parameters were not normally distri­buted, with the Shapiro-Wilk test *P*-value being <0.05. Thus, bilateral asymmetry and sex differences were tested through parametric or non-parametric approaches.

Differences between right and left femora were examined for all three measurements. *AC* parameters did not demonstrate a statistically significant diffe­rence between right and left (*t*(295) = −1.79, *P* > 0.05), as indicated by a paired samples t-test. Wilcoxon signed-rank tests for analysis of *AB* and *BC* measurements produced scores that were approximately symmetrically distributed, as assessed by histograms with a normal fitted curve. A non-statistically significant difference (*P* > 0.05) was observed between the right and left *AB* and *BC* parameters (*z* = 1.811 and *z* = 1.823, respectively).

Sex differences were examined using the independent samples Mann-Whitney *U* Test (Table 3). All six variables produced distributions that were similar between males and females, as assessed by visual inspection. All parameters were shown to be statistically significant at the <0.001 level. The SDI was calculated as described earlier, with the highest percentage values being produced by the left *AB* and right *AC* parameters.

**Table 3.** Results from Mann-Whitney *U* tests for sex difference and SDI for each parameter.

Parameter Side		Mean rank		*U*	*z* value	*P*-value	SDI
		Male	Female				
AB	R	199.28	90.36	2 878.00	−10.920	<0.001	11.99
	L	197.93	91.91	3 092.50	−10.636	<0.001	14.09
BC	R	212.12	75.66	849.50	−13.686	<0.001	13.05
	L	212.47	75.26	794.50	−13.761	<0.001	11.83
AC	R	205.47	83.27	1 900.00	−12.256	<0.001	14.27
	L	206.11	82.54	1 799.50	−12.392	<0.001	13.37

SDI: sexual dimorphism index; R: right; L: left.

The assumptions for DF analysis were tested and the data indicated suitability for this analysis through the assessment of sample size, histograms, normal Q-Q plots, and skewness and kurtosis scores [23–25]. In order to apply the formulae in a real case scenario, the respective variable measurements need to be inserted into the provided formulae. The score obtained from the discriminant function must be compared with the provided sectioning point. Score values over the sectioning point indicate that the individual is classified as a male and scores under this value indicate that the individual is classified as a female [20].

Six univariate DF equations were generated, one for each of the right and left variables collected (Table 4). The univariate DF models produced correct classification percentages ranging from the lowest (78.4%) for the right and left *AB* to the highest (90.2%) for the right *BC* (Table 5).

**Table 4.** Univariate and multivariate DF for right and left femora.

Parameter	Function	Variables	Unstd. coeff.	Constant	Wilk’s Lambda	F-ratio	Effect size	Male/female centroids	Sectioning point
Univariate DF	F1	R_AB	0.411	−12.459	0.615	183.773*	0.38	M (0.736)/F (−0.843)	−0.0535
	F2	R_BC	0.302	−17.568	0.362	517.623*	0.63	M (1.236)/F (−1.415)	−0.0895
	F3	R_AC	0.279	−13.085	0.529	261.925*	0.47	M (0.879)/F (−1.007)	−0.0640
	F4	L_AB	0.406	−12.348	0.625	176.140*	0.37	M (0.721)/F (−0.825)	−0.0520
	F5	L_BC	0.303	−17.618	0.355	534.311*	0.64	M (1.256)/F (−1.438)	−0.0910
	F6	L_AC	0.289	−13.557	0.516	275.531*	0.48	M (0.902)/F (−1.032)	−0.0652
Multivariate DF only R	F7	R_AB	0.124	−19.455	0.333	321.65*	0.67	M (1.318)/F (−1.509)	−0.0955
		R_AC	0.055	–	–	–	–	–	–
		R_BC	0.226	–	–	–	–	–	–
	F8	R_AB	0.223	−16.233	0.447	235.904*	0.55	M (1.036)/F (−1.186)	−0.0750
		R_AC	0.202	–	–	–	–	–	–
	F9	R_AB	0.133	−19.119	0.339	316.697*	0.66	M (1.300)/F (−1.488)	−0.0940
		R_BC	0.260	–	–	–	–	–	–
	F10	R_BC	0.257	−18.091	0.352	305.601*	0.64	M (1.263)/F (−1.446)	−0.0915
		R_AC	0.067	–	–	–	–	–	–
Multivariate DF only L	F11	L_AB	0.123	−19.686	0.325	328.305*	0.67	M (1.341)/F (−1.535)	−0.0970
		L_AC	0.063	–	–	–	–	–	–
		L_BC	0.223	–	–	–	–	–	–
	F12	L_AB	0.221	−16.858	0.433	245.526*	0.57	M (1.067)/F (−1.221)	−0.0770
		L_AC	0.216	–	–	–	–	–	–
	F13	L_AB	0.128	−19.196	0.333	322.108*	0.66	M (1.318)/F (−1.509)	−0.0955
		L_BC	0.263	–	–	–	–	–	–
	F14	L_BC	0.256	−18.220	0.345	311.637*	0.65	M (1.283)/F (−1.469)	−0.0930
		L_AC	0.070	–	–	–	–	–	–

Unstd. Coeff.: unstandarised coefficients; *P < 0.001; DF: discriminant function; R: right; L: left.

**Table 5.** Original and cross-validated number of individuals and percentages of correct classification.

Functions	Original correct group membership					Cross-validated correct group membership				
	Males (N=158)		Females (N=138)		Total	Males (N=158)		Females (N=138)		Total
	*n*	%	*n*	%	%	*n*	%	*n*	%	%
F1	126	79.7	106	76.8	78.4	126	79.7	106	76.8	78.4
F2	145	91.8	122	88.4	90.2	145	91.8	122	88.4	90.2
F3	128	81.0	122	88.4	84.5	128	81.0	122	88.4	84.5
F4	125	79.1	107	77.5	78.4	125	79.1	107	77.5	78.4
F5	141	89.2	123	89.1	89.2	141	89.2	123	89.2	89.2
F6	131	82.9	120	87.0	84.8	131	82.9	120	87.0	84.8
F7	146	92.4	126	91.3	91.9	146	92.4	126	91.3	91.9
F8	139	88.0	120	87.0	87.5	138	87.3	120	87.0	87.2
F9	145	91.8	125	90.6	91.2	145	91.8	125	90.6	91.2
F10	145	91.8	126	91.3	91.6	145	91.8	125	90.6	91.2
F11	147	93.0	127	92.0	92.6	146	92.4	127	92.0	92.2
F12	136	86.1	122	88.4	87.2	136	86.1	122	88.4	87.2
F13	144	91.1	127	92.0	91.6	143	90.5	127	92.0	91.2
F14	144	91.1	125	90.6	90.9	143	90.5	124	89.9	90.2

The results of the DF analysis for the right and left femora are shown in Tables 4 and 5. The first multivariate DF formulae were generated by manually entering all the measurements from the right femur (Table 4). Three of the four DF models (F7, F9, and F10) showed a slight increase in the percentage of correct classification as compared with the classification accuracy reported by the univariate DF formulae (Table 5). From the multivariate DF analysis performed on parameters collected from the left femur, the highest percentage of original and cross-validated correct classification was obtained with model F11, which includes the three parameters (*AB*, *BC*, and *AC*) (Tables 4 and 5). Note that stepwise DF for all the measurements from the right and left sides selected left *AB*, *BC*, and *AC* as the most optimal combination. This DF model was already developed in model F11.

Posterior probabilities for males, females, and total sample for the formulae reporting around 90% or higher original and cross-validated accuracy are shown in Table 6. The percentage of correctly classified individuals by each formula is represented as percentages indicating the proportion of the sample assigned to the correct group under different posterior probability thresholds. For all formulae, more than 81% of individuals were correctly classified with posterior probabilities over 0.80, and over 64% of the samples were correctly classified with posterior probabilities of 0.95 or higher. In summary, our results demonstrated sex difference in the sample under study with 7 out of the 14 formulae generated providing an overall original and cross-validated correct sex classification of over 90%.

**Table 6.** Posterior probabilities (PP%) for F2, F5, F7, F9, F10, F11, F13, and F14 for males, females, and total samples.

PP%	Males				Females				Total			
	>60	>80	>90	>95	>60	>80	>90	>95	>60	>80	>90	>95
F2	92.41	81.37	75.17	64.82	98.38	92.62	81.96	71.31	95.13	86.51	78.27	67.79
F5	97.16	85.10	78.72	70.21	99.18	95.12	81.30	69.91	98.10	89.77	79.92	70.05
F7	97.26	89.72	78.08	69.17	100.00	88.88	84.92	74.60	98.52	89.34	81.25	71.69
F9	97.93	88.27	77.24	67.58	98.40	90.40	82.39	70.39	98.15	89.25	79.62	68.88
F10	96.55	86.21	74.48	69.65	96.82	90.77	82.54	73.81	96.67	88.19	78.22	71.58
F11	96.59	85.71	79.59	70.06	98.42	92.63	85.03	76.77	97.44	89.05	82.11	72.92
F13	95.83	86.80	80.55	72.91	96.06	91.33	81.88	74.80	95.94	88.92	81.18	73.43
F14	96.52	88.19	77.08	70.13	98.40	93.60	82.39	72.00	97.39	90.70	79.55	71.00

## Discussion

Estimating sex from human remains is part of the identification routine performed by forensic anthropologists. Because forensic identification methods are subject to scrutiny, the choice of technique must rely on population proximity and accuracy rates that allow the practitioner to fulfil the evidentiary standards [26]. Additionally, both the available skeletal element and its state of preservation need to be thoroughly considered to choose the best method. If intact, the pelvis is considered the most sexually dimorphic biological indicator in the skeleton [1]. Morphoscopic analysis of the skull and mandible is considered the second choice when the pelvis is not available, although this is dependent on a number of traits and the provenance of the remains [27, 28]. However, issues arising from the intrinsic subjectivity of the morphological approach in relation to the observer’s experience, training, and lack of precise instructions and diagrams, among others, have been reported [29, 30].

Alternatively, the metric approach for sex estimation relies on the collection of measurements from specific skeletal elements by identifying anatomical landmarks and applying a quantitative approach. DF and binary logistic regression are commonly used to determine if the subject is male or female [31]. Several skeletal elements may be considered for metric sex estimation. Published studies have provided different degrees of accuracy and presented similar issues as the morphological assessment, such as skeletal element availability and population-specific size-related dimorphism [7, 32, 33]. Although considered more objective with intra- and inter-observer error being less of a concern, metric techniques are not exempt from drawbacks. Adams and Byrd [34] tested 22 postcranial measurements on observers with 0 to more than 10 years of training on handling problems related to not only parameter definition discrepancies and difficulty in anatomical landmark identification, but also to observers’ years of experience and equipment used. Djorojevic et al. [12] tested both intra- and inter-observer errors for Purkait’s [11] parameters, concluding that the method has high reliability based on low observer error results. Our study tested intra- and inter-observer errors on the virtual femoral measurements through TEM analysis, with all rTEM values falling within the levels of acceptance. This confirmed the reliability of the parameters, corroborating other studies conducted on virtual data [33, 35–36]. Interestingly, the lowest R scores were reported for *BC* parameters, with 8% of the variance related to measurement error for right and left intra-observer error. The lowest R value corresponded to left *BC*, with 14% of the variance from inter-observer scoring error. Factors such as the nature of the parameter and landmark identification through 3D images may have had an impact on *BC* results. Further virtual studies on Purkait’s method [11] should be performed to obtain more data and draw appropriate conclusions. Furthermore, other issues, such as the segmentation protocol as a possible source of bias, might be taken into consideration in future observer error testing [37].

The present research was conducted on 296 adult Turkish individuals by virtually applying the method proposed by Purkait [11]. Our results indicated that no bilateral asymmetry exists between right and left femora based on the measurements collected, which is consistent with other studies [11, 13]. However, research focusing on orthopaedics has included more metric data that indicate contradictory side-to-side differences for specific anatomical segments within the proximal femoral area [38, 39]. As expected, all parameters demonstrated statistically significant differences between the sexes, with males having higher values than females. Both the right and left sides were tested and used separately to generate the population-specific sex estimation method based on the Turkish samples in this study. We recommend the use of univariate DF models that apply right and left *BC* measurements to ensure accuracies higher than 89%. Depending on bone preservation, all three parameters from the right femur or the combination of right *AB* and *BC* or right *BC* and *AC*, separately, can be used to reach a correct sex classification of 91%. If only the left femur is available, all the parameters combined are recommended to obtain the most accurate classification produced in the present research (92%). Overall, our classification results are slightly higher when compared with the errors reported by the original study, with the remaining three published papers on the application of Purkait’s triangle method reporting cross-validated correct classifications ranging from 54.7% to 85.5% [12–14] (Table 7). When analysing the comparative table, it should be taken into consideration that Purkait [11] used 200 males and 80 females. This is an unbalanced sample collection and should be considered when interpreting the results.

**Table 7.** Studies on proximal femur triangle methods indicating samples and accuracies rates.

Study	Population (*N*: *n*_male, *n*_female)	Cross-validated % range	
		Univariate DF	Multivariate DF
Purkait [11]	Indian (280: 200, 80)	62.5–84.3^a^	85.4^a^–87.5
Brown et al. [14]	European American and African American (200: 100, 100)	69.0–85.5	74.0–87.0
Anastopoulou et al. [13]	Greeks (203: 112, 91)	54.7–77.3	73.4–76.2
Djorojevic et al. [12]	Spanish (186: 77, 109)	76.9–85.5	81.7–83.3
The present study	Turkish (296: 158, 138)	78.4–9.2	87.2–92.2

^a^Average correct classification; *N*: total number of individuals; *n*: sex subsamples; DF: discriminant function.

Differences in the SDI can be observed between populations in relation to Purkait’s triangle method [11, 13, 14]. Although the representation of each sex in the existing studies must be taken into consideration when comparing the present sample set with the published data, overall, the Turkish population shows an SDI for *BC* similar to those of Indian and European American populations. Greater values than that reported by the Greek population might explain the higher classification accuracies for the Turkish samples compared with the Athens samples. The remaining measurements do show diffe­rences, with *AB* showing the highest SDI for European Americans and the lowest for Indians, while the inverse result was observed for *AC*. In the Turkish sample, *AC* demonstrated a higher SDI than *BC*, which was also observed in the Greek samples [13].

Interestingly, the intertrochanteric apex distance (*BC*) was selected by DF analysis as the best single parameter to discriminate between sexes in our study, as well as in Purkait [11], Brown et al. [14], Anastopoulou et al. [13], and Djorojevic et al. [12]. The iliofemoral ligament attaches superiorly to the intertrochanteric line and is the largest ligament in the human body [40]. It is expected that this measurement will better represent sexual dimorphism being more robust in males and an area of upper body weight transmission [12]. Research has focused on this specific segment of the proximal femur because of its relevance to hip fracture and its relationships with age, sex, and related diseases such as osteoporosis [41, 42]. Previous studies noted size-related sexual dimorphism in proximal femur parameters, such as neck and head diameters, with accuracies around 89% for single measurements [7, 43, 44]. Although common in humans and animals [5], sexual dimorphism presents to various degrees in different populations. Thus, femoral sex estimation population-specific standards have been conducted to understand size and shape differences and to validate and test accuracy rates of correct sex classification for forensic identification [45–47]. Reported metric differences between Indians and other populations indicated the possible impact of sex, ethnicity, genetics, diet, and physical activity, among others factors affecting the accuracy rates obtained [49, 50]. Specifically, sexual dimorphism inter-population variation in relation to Purkait’s triangle has been previously shown [12]. Therefore, the development of Turkish population standards for this method was recommended.

The Turkish population has been studied for sex estimation and presented different levels of correct classification using the skulls of 400 individuals (77%–89%) [50], sternums of 443 individuals (56.4%–86.1%) [51], scapulas of 152 individuals (74.3%–96.1%) [37], tibias of 203 individuals (77.8%–84.7%) [36], calcaneus of 428 individuals (89.9%–100%) [52], and clavicles of 152 individuals (75%–89%). Gulhan et al. [53] conducted a sex estimation CT study on 200 modern adult Turkish individuals by applying 13 parameters collected from the femur. They obtained a 63.5% to 88.0% accuracy rate for univariate DF formulae and a 91% accuracy rate for a three-parameter multivariate DF formula. The results provided by the DF analysis performed on parameters from the femoral proximal area were consistent with the reported classification accuracies. They provided slightly higher correct classification for single parameters (F2 and F5) than the ones reported by Gulhan et al. [53].

Virtual anthropology is an ever evolving field within biological anthropology and is used as a tool in forensic anthropology as means for sex and age estimation, trauma analysis, and commingled remains analysis, among other uses [54–56]. The feasibility of using virtual data in place of the traditional dry bone macroscopic observation has been tested for morphological and metric assessments [35, 54]. Its advantages over the traditional approach are related to the observation of all structures within the bone, the remote accessibility of the data, and the simultaneous analysis of size and shape. This can widen the research opportunities within the field [57, 58]. 3D-CT images were used as a tool in the present study and allowed the authors to develop a population-specific method for sex estimation surpassing the lack of an osteological collection. Although previous stu­dies have confirmed the accuracy of metric virtual data compared with dry bone measurements, further validation and standardisation are still required to confirm the application of virtual data to dry bones, and vice versa [58, 59].

## Conclusion

Because sex estimation is a required aspect of generating a biological profile for an unidentified individual, population-specific standards are necessary to ensure they are assigned to the correct group. In this study, we presented a sex estimation method for Turkish individuals through the application of Purkait’s method [11]. The results demonstrate the overall reliability of this method through virtual data, with our accurate classification rates being comparable to previous analyses performed on dry proximal femora. Additionally, it can potentially be applied to forensic identification because it provided an overall accuracy around 90% for most proximal femur parameters and posterior probabilities of over 95% for over 67% of the sample examined. As other populations have been assessed using the same technique, forensic anthropologists are advised to apply specific-population methods if the geographical origin of the unidentified individual is known, reducing the errors of sex misclassification.

## Authors’ contributions

Suna Ors, Ercan Inci and Oguzhan Ekizoglu collected and analysed the data. Julieta G. García-Donas and Elena Kranioti analysed the data, wrote and edited the manuscript. Oguzhan Ekizoglu, Negahnaz Moghaddam and Silke Grabherr wrote and edited the manuscript. All authors assisted with writing and editing the manuscript and approved the final version.

## References

[1] Krogman W, Isçan M. The human skeleton in forensic medicine. 2nd ed. Springfield (IL): Charles C. Thomas; 1986.

[2] Ubelaker DH, Shamlou A, Kunkle A. Contributions of forensic anthropology to positive scientific identification: a critical review. Forensic Sci Res. 2019;4:45–50. 30915416 10.1080/20961790.2018.1523704 PMC6427489

[3] Isçan M, Loth S, Wright R. Age estimation from the rib by phase analysis: white females. J Forensic Sci. 1984;30:853–863. 4031812

[4] Isçan M, Loth S, Wright R. Age estimation from the rib by phase analysis: white males. J Forensic Sci. 1984;29:1094–1104. 6502109

[5] Nikitovic D. Sexual dimorphism (humans). Int Encycl Biol Anthropol. 2018;1–4.

[6] Meek SB, Peckmann TR, Meek S, et al. Sex estimation using diagonal diameter measurements of molar teeth in African American populations. J Forensic Leg Med. 2015;36:70–80. 26408392 10.1016/j.jflm.2015.09.001

[7] Spradley MK, Jantz RL. Sex estimation in forensic anthropology: skull *versus* postcranial elements. J Forensic Sci. 2011;56:289–296. 21210801 10.1111/j.1556-4029.2010.01635.x

[8] Krishan K, Chatterjee PM, Kanchan T, et al. A review of sex estimation techniques during examination of skeletal remains in forensic anthropology casework. Forensic Sci Int. 2016;261:165.e1–e8. 26926105 10.1016/j.forsciint.2016.02.007

[9] Alunni-Perret V, Staccini P, Quatrehomme G. Sex determination from the distal part of the femur in a French contemporary population. Forensic Sci Int. 2008;175:113–117. 17624707 10.1016/j.forsciint.2007.05.018

[10] Alunni V, Du Jardin P, Nogueira L, et al. Comparing discriminant analysis and neural network for the determination of sex using femur head measurements. Forensic Sci Int. 2015;253:81–87. 26093772 10.1016/j.forsciint.2015.05.023

[11] Purkait R. Triangle identified at the proximal end of femur: a new sex determinant. Forensic Sci Int. 2005;147:135–139. 15567617 10.1016/j.forsciint.2004.08.005

[12] Djorojevic M, Roldán C, Botella M, et al. Estimation of Purkait’s triangle method and alternative models for sex assessment from the proximal femur in the Spanish population. Int J Legal Med. 2016;130:245–251. 25951948 10.1007/s00414-015-1201-7

[13] Anastopoulou I, Eliopoulos C, Valakos ED, et al. Application of Purkait’s triangle method on a skeletal population from southern Europe. Forensic Sci Int. 2014;245:203.e1–e4. 25459271 10.1016/j.forsciint.2014.10.005

[14] Brown R, Ubelaker D, Schanfield M. Evaluation of Purkait’s triangle method for determining sexual dimorphism. J Forensic Sci. 2007;52:553–556. 17456081 10.1111/j.1556-4029.2007.00423.x

[15] Kranioti EF, Vorniotakis N, Galiatsou C, et al. Sex identification and software development using digital femoral head radiographs. Forensic Sci Int. 2009;189:113.e1–e7. 19443150 10.1016/j.forsciint.2009.04.014

[16] Mostafa EM, El-Elemi AH, El-Beblawy MA, et al. Adult sex identification using digital radiographs of the proximal epiphysis of the femur at Suez Canal University Hospital in Ismailia, Egypt. Egypt J Forensic Sci. 2012;2:81–88.

[17] Ulijaszek SJ, Kerr DA. Anthropometric measurement error and the assessment of nutritional status. Br J Nutr. 1999;44:165–177. 10.1017/s0007114599001348 10655963

[18] Ricklan D, Tobias P. Unusually low sexual dimorphism of endocranial capacity in a Zulu cranial series. Am J Phys Anthropol. 1986;71:285–293. 3812650 10.1002/ajpa.1330710304

[19] Klecka W. Discriminant analysis. Thousand Oaks (CA): Sage Publications, Inc.; 1980. (Quantitative applications in the social sciences; 19).

[20] Hair J, Black W, Babin B, et al. Multiple discriminant analysis. In: Multivariate Data Analysis. 7th ed. Essex (UK): Pearson Education Limited; 2018. p. 471–547.

[21] Campbell N. Multivariate analysis in biological anthropology: some further considerations. J Hum Evol. 1978;7:197–203.

[22] Kranioti E, Apostol MA. Sexual dimorphism of the tibia in contemporary Greeks, Italians, and Spanish: forensic implications. Int J Legal Med. 2015;129:357–363. 25047696 10.1007/s00414-014-1045-6

[23] Tabachnick B, Fidell L. Using multivariate statistics. 6th ed. Boston (MA): Pearson Education Limited; 2013.

[24] Sheskin D. Handbook of parametric and nonparametric statistical procedures. 5th ed. Boca Raton (FL): Chapman & Hall/CRC Press; 2011.

[25] Kim H-Y. Statistical notes for clinical researchers: assessing normal distribution (2) using skewness and kurtosis. Restor Dent Endod. 2013;38:52–54. 23495371 10.5395/rde.2013.38.1.52 PMC3591587

[26] Christensen AM, Crowder CM. Evidentiary standards for forensic anthropology. J Forensic Sci. 2009;54:1211–1216. 19804520 10.1111/j.1556-4029.2009.01176.x

[27] Williams BA, Rogers T. Evaluating the accuracy and precision of cranial morphological traits for sex determination. J Forensic Sci. 2006;51:729–735. 16882212 10.1111/j.1556-4029.2006.00177.x

[28] Walker PL. Sexing skulls using discriminant function analysis of visually assessed traits. Am J Phys Anthropol. 2008;136:39–50. 18324631 10.1002/ajpa.20776

[29] Bruzek J. A method for visual determination of sex, using the human hip bone. Am J Phys Anthropol. 2002;117:157–168. 11815949 10.1002/ajpa.10012

[30] Walrath DE, Turner P, Bruzek J. Reliability test of the visual assessment of cranial traits for sex determination. Am J Phys Anthropol. 2004;125:132–137. 15365979 10.1002/ajpa.10373

[31] Iscan MY, Steyn M. The human skeleton in forensic medicine. 3rd ed. Springfield (IL): Charles C. Thomas; 2013. p. 143–194.

[32] Yaşar Işcan M, Steyn M. Craniometric determination of population affinity in South Africans. Int J Legal Med. 1999;112:91–97. 10048665 10.1007/s004140050208

[33] Kranioti EK, García-Donas JG, Almeida Prado PS, et al. Sexual dimorphism of the tibia in contemporary Greek-Cypriots and Cretans: forensic applications. Forensic Sci Int. 2017;271:129.e1–129.e7. 10.1016/j.forsciint.2016.11.018 27919515

[34] Adams BJ, Byrd JE. Interobserver variation of selected postcranial skeletal measurements. J Forensic Sci. 2002;47:15550J. 12455639

[35] Decker SJ, Davy-Jow SL, Ford JM. Virtual determination of sex: metric and nonmetric traits of the adult pelvis from 3D computed tomography models. J Forensic Sci. 2011;56:31–36. 10.1111/j.1556-4029.2011.01803.x 21595690

[36] Ekizoglu O, Er A, Bozdag M, et al. Sex estimation of the tibia in modern Turkish: a computed tomo­graphy study Oguzhan. J Leg Med. 2016; 23:89–94. 10.1016/j.legalmed.2016.10.004 27890111

[37] Er A, Kacmaz IE, Ekizoglu O. Sex estimation of the scapula using 3D imaging in a modern Turkish population. Rechtsmedizin. 2020;30:209–218.

[38] Young E, Gebhart J, Cooperman D, et al. Are the left and right proximal femurs symmetric? Clin Orthop Relat Res. 2013;471:1593–1601. 23179124 10.1007/s11999-012-2704-x PMC3613562

[39] Dimitriou D, Tsai T, Yue B, et al. Side-to-side variation in normal femoral morphology: 3D CT analysis of 122 femurs. Orthop Traumatol Surg Res. 2016;102:91–97. 26867707 10.1016/j.otsr.2015.11.004

[40] Gray H, Standring S, Ellis H, et al. Musculoskeletal system. In: Standing S, editor. Graýs anatomy. 39th ed. New York (NY): Elsevier Churchill Livingstone; 2005. p. 83–135.

[41] Fox KM, Magaziner J, Hebel JR, et al. Intertrochanteric *versus* femoral neck hip fractures: differential characteristics, treatment, and sequelae. J Gerontol Med Sci. 1999;54:635–640. 10.1093/gerona/54.12.m635 10647970

[42] Tsai S, Lin CJ, Tzeng Y, et al. Risk factors for cut-out failure of Gamma3 nails in treating unstable intertrochanteric fractures: an analysis of 176 patients. J Chin Med Assoc. 2017;80:587–594. 28601630 10.1016/j.jcma.2017.04.007

[43] Mall G, Graw M, Gehring K, et al. Determination of sex from femora. Forensic Sci Int. 2000;113:315–321. 10978643 10.1016/s0379-0738(00)00240-1

[44] Frutos L. Brief communication: sex determination accuracy of the minimum supero-inferior femoral neck diameter in a contemporary rural Guatemalan population. Am J Phys Anthropol. 2003;122:123–126. 12949832 10.1002/ajpa.10227

[45] Albanese J. A metric method for sex determination using the hipbone and the femur. J Forensic Sci. 2020;48:1–11. 12664981

[46] Slaus M, Strinovic D, Skavic J, et al. Discriminant function sexing of fragmentary and complete femora: standards for contemporary Croatia. J Forensic Sci. 2020;48:1–4. 12762518

[47] Steyn M, Işcan MY. Sex determination from the femur and tibia in South African whites. Forensic Sci Int. 1997;90:111–119. 9438371 10.1016/s0379-0738(97)00156-4

[48] Krishan K, Sharma A. Estimation of stature from dimensions of hands and feet in a North Indian population. J Forensic Leg Med. 2007;14:327–332. 17239650 10.1016/j.jcfm.2006.10.008

[49] Ahmed AA. Estimation of sex from the lower limb measurements of Sudanese adults. Forensic Sci Int. 2013;229:169.e1–e7. 23642728 10.1016/j.forsciint.2013.04.005

[50] Ekizoglu O, Hocaoglu E, Inci E, et al. Assessment of sex in a modern Turkish population using cranial anthropometric parameters. Leg Med (Tokyo). 2016;21:45–52. 27497333 10.1016/j.legalmed.2016.06.001

[51] Ekizoglu O, Hocaoglu E, Inci E, et al. Sex estimation from sternal measurements using multidetector computed tomography. Rechtsmedizin. 2014;93:e240. 10.1097/MD.0000000000000240 PMC4602791 25501090

[52] Ekizoglu O, Inci E, Bakirtas F, et al. Sex estimation in a contemporary Turkish population based on CT scans of the calcaneus. Forensic Sci Int. 2017;279:310.e1–e6. 28912044 10.1016/j.forsciint.2017.07.038

[53] Gulhan O, Harrison K, Kiris A. A new computer-tomography-based method of sex estimation: development of Turkish population—specific standards. Forensic Sci Int. 2015;255:2–8. 26250530 10.1016/j.forsciint.2015.07.015

[54] Grabherr S, Cooper C, Ulrich-Bochsler S, et al. Estimation of sex and age of “virtual skeletons”—a feasibility study. Eur Radiol. 2009;19:419–429. 18766348 10.1007/s00330-008-1155-y

[55] Urbanova P, Ross A, Jurda M, et al. The virtual approach to the assessment of skeletal injuries in human skeletal remains of forensic importance. J Forensic Leg Med. 2017;49:59–75. 28586732 10.1016/j.jflm.2017.05.015

[56] Karell MA, Langstaff HK, Halazonetis DJ, et al. A novel method for pair-matching using three-dimensional digital models of bone: mesh-to-mesh value comparison. Int J Legal Med. 2016;130:1315–1322. 26966098 10.1007/s00414-016-1334-3 PMC4976056

[57] Weber GW, Schäfer K, Prossinger H, et al. Virtual anthropology: the digital evolution in anthropological sciences. J Physiol Anthropol Appl Human Sci. 2001;20:69–80. 10.2114/jpa.20.69 11385941

[58] Garvin HM, Stock MK. The utility of advanced imaging in forensic anthropology. Acad Forensic Pathol. 2016;6:499–516. 31239924 10.23907/2016.050 PMC6474549

[59] Franklin D, Cardini A, Flavel A, et al. Concordance of traditional osteometric and volume-rendered MSCT interlandmark cranial measurements. Int J Legal Med. 2013;127:505–520. 23052442 10.1007/s00414-012-0772-9

